# Performance of Halloysite-Mg/Al LDH Materials for Aqueous As(V) and Cr(VI) Removal

**DOI:** 10.3390/ma12213569

**Published:** 2019-10-31

**Authors:** Jakub Matusik, Jakub Hyla, Paulina Maziarz, Karolina Rybka, Tiina Leiviskä

**Affiliations:** 1 Department of Mineralogy, Petrography and Geochemistry, Geophysics and Environmental Protection, Faculty of Geology, AGH University of Science and Technology, Mickiewicza 30, 30-059 Krakow, Poland; kuba.251@wp.pl (J.H.); pmaziarz@agh.edu.pl (P.M.); krybka@agh.edu.pl (K.R.); 2 Chemical Process Engineering, University of Oulu, P.O. Box 4300, 90570 Oulu, Finland; tiina.leiviska@oulu.fi

**Keywords:** halloysite, layered double hydroxide, arsenates, chromates, adsorption, ion exchange

## Abstract

This research focused on the investigation of layered double hydroxide (LDH)/halloysite materials’ adsorption efficiency and mechanisms in reactions with aqueous As(V) and Cr(VI) in a broad pH range. The materials consisting of Mg/Al LDH and halloysite were synthesized using both direct precipitation and physical mixing methods. The XRD, FTIR, DTA, SEM and XPS methods were used to evaluate the quality of the obtained materials and get insight into removal mechanisms. The XRD, FTIR and DTA confirmed LDH formation and showed the dominating presence of intercalated carbonates in the LDH structure. The SEM of the materials revealed characteristic agglomerates of layered LDH particles deposited on halloysite tubular forms. The raw LDH phases showed high removal efficiency of both As(V) and Cr (VI) for initial pH in the range of 3–7. In the studied concentration range the materials containing 25 wt % of LDH exhibited a removal efficiency very similar to the raw LDH. In particular, the halloysite presence in the materials’ mass had a positive effect in the reactions with As(V), which was removed by chemisorption. At a low pH the LDH component underwent partial dissolution, which lowered the adsorption efficiency. Apart from the anion exchange mechanism at a low pH the Cr(VI) was removed via formation of MgCrO_4_ with Mg (II) being released from the LDH structure. The XPS spectra for As(V) did not show changes in oxidation state in the reactions. In turn, a partial reduction of Cr(VI) to Cr(III) was observed, especially at a high pH. The use of materials composed of two different minerals is promising due to reduction of costs as well as prevention of adsorbent swelling. This opens the possibility of its use in dynamic adsorption flow through systems.

## 1. Introduction

Along with the intense and rapid development of industry, one of the major concerns is the generation of large volumes of wastewater that would affect the surrounding environments and subsequently human health. Industrial wastewaters often exhibit a complex chemical composition, which causes difficulties in selecting appropriate and effective treatment technologies. Methods—generally physical, chemical or biological—typically include different types of processes, such as flotation, filtration, reverse osmosis, precipitation or adsorption [1]. During the past decade, various conventional and innovative removal approaches were studied and developed [2,3,4,5,6,7,8,9,10,11,12,13]. Among them, the adsorption technique is generally considered to be an effective, low-cost and versatile method capable of removing both cations and anions. In recent years, a particular research focus is on synthesis and application of new hybrid adsorbents [14,15,16,17,18]. A combination of phases exhibiting various properties can result in an increased stability and adsorption efficiency, which can lead to new possibilities.

In recent years, the layered double hydroxide (LDH)-based materials are extensively studied [19,20,21,22,23,24,25]. The LDH are a class of two-dimensional lamellar anionic clays, composed of positively charged brucite-like layers and charge-balancing anions in the interlayer galleries [26]. The general chemical formula can be described as: [M^II^_1−x_ M^III^_x_ OH_2_]^x+^ [A^n−^]_x/n_ y H_2_O, where M^II^ and M^III^ are divalent (e.g., Ca^2+^, Mg^2+^, Mn^2+^, Fe^2+^, Co^2+^, Ni^2+^, Cu^2+^ or Zn^2+^) and trivalent (e.g. Al^3+^, Fe^3+^) metal cations and the A^n−^ represents exchangeable interlayer anions (e.g., OH^−^, Cl^−^, SO_4_^2−^, NO_3_^−^ and CO_3_^2−^). The features of LDH phases, such as ease of synthesis, flexible chemical composition, layered structure and large surface area, contribute to their potential application in catalysis, electrochemistry, polymer chemistry, biomedicine and wastewater treatment [7,27,28,29,30,31,32,33,34,35]. However, due to poor dispersity and low chemical stability in acidic conditions it is essential to develop LDH-based hybrids [36,37,38]. Such an approach can reduce production costs of the adsorbent where the active LDH component is in sufficient amount for the efficient removal of the pollutant, and at the same time it is stabilized by the support material. It is possible and desirable that the support can play a synergistic role in the treatment process. While LDH-based hybrids have been mostly tested in laboratory-scale batch experiments, it is essential to expand the study to real systems. The application of the halloysite support for the LDH should result in increased physical and chemical stability as well as a reduction of undesirable swelling properties. The latter poses a limitation of using pure LDH phases in dynamic adsorption conditions [37]. In our recent work we have summarized findings on Mg-Fe LDH derived from chemicals or magnesite and its composites with halloysite [38]. The materials in uncalcined and calcined form showed high efficiency in the removal of Cr(VI) and SO_4_^2−^ from real wastewaters.

In this study, the hybrid materials of Mg-Al LDH and halloysite were synthesized. In the experiment, 5, 15 and 25 wt % loading of LDH on halloysite were used. The materials were prepared both by physical mixing of earlier prepared LDH with halloysite and direct precipitation of the LDH phase on the halloysite surface. The adsorption properties of the resulting materials were studied in reactions of As(V) and Cr(VI) removal. Most importantly, the removal mechanisms were investigated using solid-state analytical techniques, in particular including X-ray photoelectron spectroscopy for oxidation state determination of As and Cr.

## 2. Materials and Methods

### 2.1. Materials

The halloysite sample (H) was obtained from the Dunino deposit (Intermark company, Gliwice, Poland). The sample contains both dehydrated halloysite-7 Å and kaolinite in a ~60:40 ratio as attested by a formamide test [39]. The mineral particles exhibit different morphologies with domination of tubes and plates. All chemical reagents were of analytical grade and distilled water was used for the experiments.

### 2.2. Synthesis of LDH and Halloysite-LDH Materials

The Mg/Al·LDH·(LDH) was synthesized by a standard co-precipitation method using MgCl_2_·6H_2_O and AlCl_3_·6H_2_O as sources of metals. The Mg:Al molar ratio of 2:1 was set by preparing an aqueous solution containing both metals with the following concentrations: 1.2 mol/L Mg^2+^ and 0.6 mol/L Al^3+^. This solution was added dropwise to a beaker containing 100 mL of 1 M NaCl with the initial pH set to 10 by a diluted NaOH aqueous solution. The forming dispersion was stirred, and the pH was constantly controlled in the range of 9–10. The final precipitate was aged for 24 h at room temperature (22 °C), then centrifuged (4500 rpm, 10 min), washed with distilled water and dried at 60 °C overnight.

The halloysite-LDH materials (H-LDH) containing 5, 15 and 25 wt % of LDH were prepared by using two different approaches: precipitation (P) and mixing (M). The obtained products were abbreviated as 5 H-LDH-P, 15 H-LDH-P, 25 H-LDH-P and 5 H-LDH-M, 15 H-LDH-M and 25 H-LDH-M, respectively. In the first synthesis route, the halloysite sample was dispersed in the 1 M NaCl solution before addition of a solution containing metals. Afterwards the procedure was identical as for the pure LDH. This procedure assumed direct precipitation of LDH on the halloysite support surface. The calculations based on reaction stoichiometry allowed to control the amount of formed LDH. In the second approach the pure LDH was physically mixed with halloysite in a dry state by grinding in a mortar.

### 2.3. Adsorption Experiments

The removal efficiency for the studied materials was examined in single-element systems containing Cr(VI) or As(V) introduced in the form of potassium chromate K_2_Cr_2_O_7_ and sodium arsenate dibasic heptahydrate Na_2_HAsO_4_·7H_2_O, respectively. In the batch experiments two initial concentrations (C_in_) of Cr(VI) or As(V) were used, 1 and 5 mmol/L, and four initial pH values (pH_in_) were tested, 1, 3, 5 and 7. The pH was controlled by using a diluted aqueous solutions of HCl or NaOH. The solid-to-liquid ratio was constant and equal to 20 g/L and the adsorbent samples were shaken for 24 h. Afterwards the samples were centrifuged, and the concentration of selected ions was analyzed in the supernatant solutions. All experiments were run in duplicates at room temperature. The errors were calculated as standard deviation from all performed measurements and were presented as error bars in Figures 5, 6, Figures S2 and S3.

The chemical composition of solutions was analyzed using the 1,5-diphenylcarbazide colorimetric method for Cr(VI) and molybdenum blue method for As(V) [40,41]. The concentrations of Cr(VI) and As(V) were calculated using the calibration curve approach with absorption measured by a UV-Vis spectrophotometer (Hitachi U-1800 instrument, Tokyo, Japan). The concentration of Mg(II) was analyzed using atomic absorption spectrometry (AAS) using the GBC SavanthAA instrument (Braeside, Australia).

### 2.4. Analytical Methods for Solid Samples

The obtained materials were characterized by X-ray diffraction (XRD), Fourier transformed infrared (FTIR) spectroscopy, scanning electron microscopy (SEM) and differential thermal analysis (DTA). The XRD patterns were obtained by using a RIGAKU Miniflex 600 diffractometer with CuKα (λ = 1.5418 Å) radiation (Tokyo, Japan). The patterns of powdered samples were recorded in the range of 2–72° 2θ with a 0.05° 2θ step. The infrared spectra were collected by KBr pellet method (1 mg sample mixed with 200 mg KBr) with a Nicolet 6700 spectrometer (Thermo Scientific, Waltham, MA, USA). For each measurement, 64 scans were collected in the range of 4000–400 cm^−1^ and a 4 cm^−1^ resolution. The SEM images were obtained using a FEI Quanta 200 FEG microscope (Hillsboro, OR, USA) under low vacuum. The samples were prepared by placing powdered material on a carbon tape. The thermal (DTA/TG) analysis of the samples was carried out using a Netzsch STA 449F3 instrument coupled with a Quadrupole Mass Spectrometer Netzsch QMS 403 (Selb, Germany). The measurements were performed using ~20 mg samples in a temperature range of 25–1000 °C (heating rate: 10 °C/min, air atmosphere). The X-ray photoelectron spectroscopy (XPS) was used for the characterization of selected samples after reaction with As(V) and Cr(VI). The spectra were recorded by a Thermo Fisher Scientific ESCALAB 250Xi spectrometer (Waltham, MA, USA) using monochromatic Al Kα radiation (1486.6 eV). The C1s peak at 285 eV was selected as the binding energy reference. The wide-scan spectra were recorded in steps of 1 eV and a pass energy of 150 eV, while high-resolution spectra with steps of 0.1 eV and a pass energy of 20 eV. The peak fitting was performed using Avantage software.

## 3. Results and Discussion

### 3.1. Characterization of Adsorbents

#### 3.1.1. XRD Results

**Figure 1:**
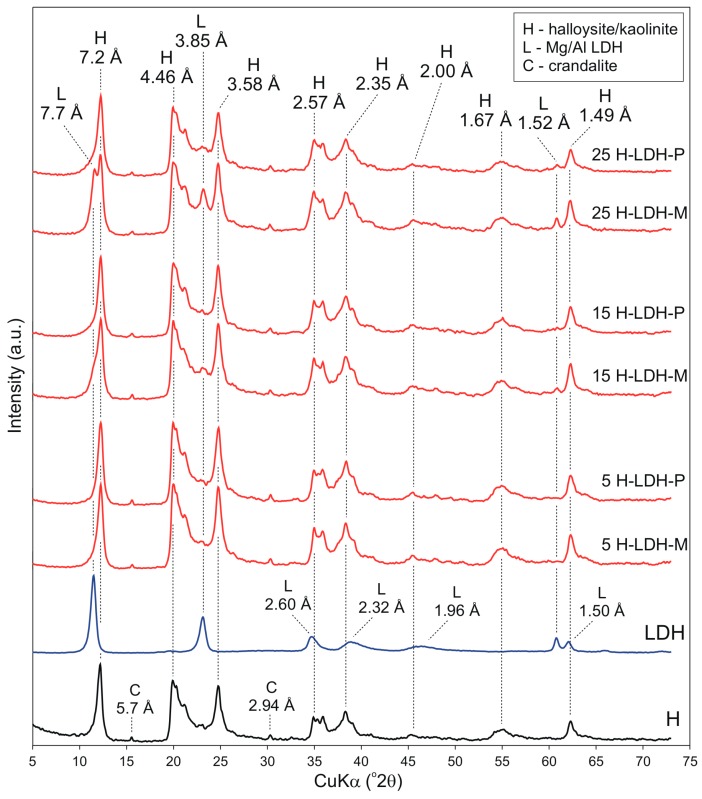
XRD patterns of halloysite sample (H), layered double hydroxide (LDH) and H-LDH materials.

The XRD pattern of H sample showed reflection at 7.20 Å characteristic for kaolin minerals (Figure 1). Additionally, reflections at 5.70 Å and 2.94 Å revealed the presence of crandalite CaAl_3_(PO_4_)_2_(OH)_5_·H_2_O in the sample, which is the by-product of the halloysite processing. The LDH formation was confirmed by appearance of reflections at 7.70 Å, 3.85 Å, 2.60 Å, 2.32 Å, 1.96 Å, 1.52 Å and 1.50 Å, characteristic for the hydrotalcite, which is a natural Mg/Al LDH (ICDD #14-191). The high crystallinity and well-ordered layered structure of LDH phase was indicated by sharp diffraction reflections. Moreover, the lack of additional reflections confirmed a high purity of the synthesized LDH. In the XRD patterns of H-LDH materials the evidences for H as well as LDH phases were observed. However distinct LDH reflections can be easily distinguished only for the H-LDH materials having 15 or 25 wt % LDH content. In case of other materials, the intensity changes as well as the broadening of reflections in regions corresponding to the LDH phase was observed. It is worth noticing that differences in the XRD patterns between M and P materials were not observed. The exception is for the 25 H-LDH-M and 25 H-LDH-P, where clearly resolved LDH and H basal reflections were observed for the first sample.

#### 3.1.2. FTIR Results

**Figure 2:**
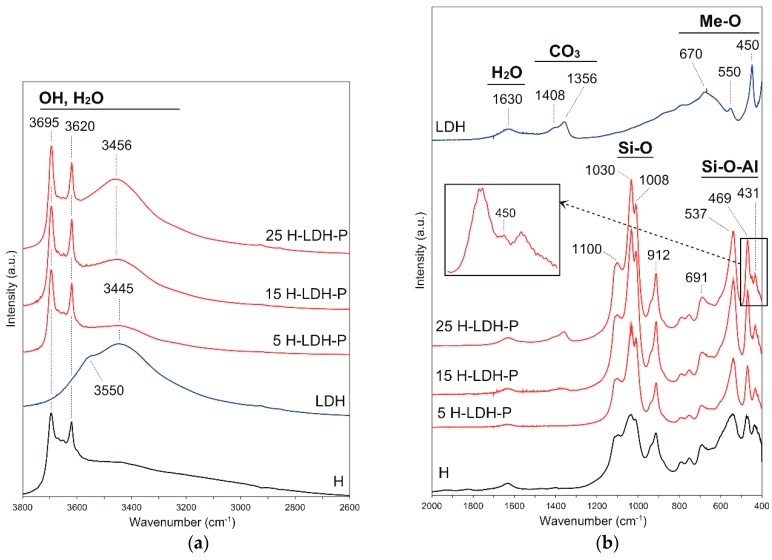
FTIR spectra of halloysite sample H, LDH and H-LDH-P materials in the (**a**) 3800–2600 cm^−1^ range and (**b**) 2000–400 cm^−1^ range.

The FTIR spectra of starting materials and the obtained materials showed bands attributed to the presence of OH groups and H_2_O in the 3700–3200 cm^−1^ region (Figure 2a). In the LDH and H-LDH materials’ spectra, bands representing stretching vibrations of OH groups in the brucite layer and interlayer water were found in the 3600–3445 cm^−1^ region. The intensities of these bands were higher for the pure LDH and material with 25 wt % of LDH, which indirectly show an increase in LDH content. The sharp bands at 3695 cm^−1^ and 3620 cm^−1^ were attributed to the inner surface and inner OH groups, respectively [42]. The band at 1630 cm^−1^ indicated the presence of intercalated water molecules in the LDH sample (Figure 2b). The presence of carbonates in the LDH sample was confirmed by bands in the region 1460–1300 cm^−1^. The bands with the maximum at 1408 and 1356 cm^−1^ represent the strong basic monodentate carbonate species [43]. However, the weak band at 876 cm^−1^ was assigned to the CO_3_^2−^ stretching mode and out-of-plane deformation mode of bicarbonates. The band at 670 cm^−1^ was attributed to the in-plane deformation mode of CO_3_^2−^ [37,44]. The weak band at 790 cm^−1^ and bands at 550 and 450 cm^−1^ represent the lattice vibrations of the Me–O type in the LDH structure. In the H sample spectrum, the bands related to the water (1630 cm^−1^) and carbonates (1405 cm^−1^) were also visible. In the regions of 1140–980 and 800–400 cm^−1^ sharp bands indicated the Si-O and Si-O-Al vibrations of the halloysite aluminosilicate framework [45]. In the spectra of H-LDH materials, bands of halloysite were overlapping bands characteristic for the LDH. However, with the increasing amount of LDH in the sample, in the region of 1460–1300 cm^−1^ the bands assigned to carbonates were more visible. The band at 450 cm^−1^ also related to the Me–O vibrations of brucite layers, was clearly visible in the 25 H-LDH-P sample and confirmed the presence of LDH in the materials’ mass.

#### 3.1.3. SEM Results

**Figure 3:**
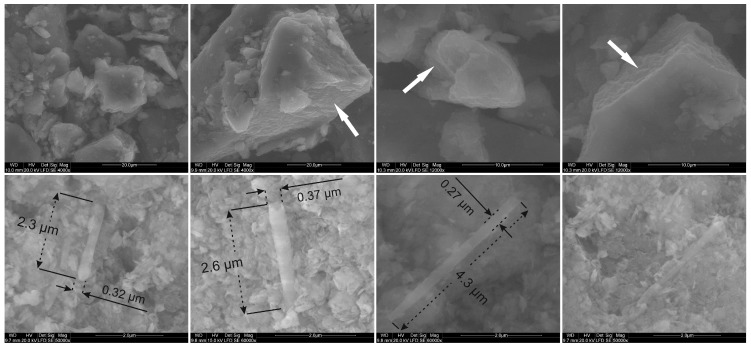
SEM microphotographs of the LDH sample (4 upper images) and 25 H-LDH-P material (4 bottom images). The white arrows indicate cross sections of the agglomerates with visible stacking of LDH particles.

The SEM images of the pure LDH phase showed particles forming agglomerates of various dimensions (Figure 3). The cross section of individual agglomerates enables to observe the characteristic layered morphology of the LDH. No clear differences in morphology were found for the materials prepared by physical mixing and precipitation. The morphology of the materials comprises of the nanometer-sized LDH particles which surround the much larger nanotubular particles of halloysite. In some cases, a characteristic “house of cards” arrangement of the LDH particles can be seen. The halloysite tubes have an approximate length varying from 2–5 µm, and outer diameter of around 0.3–0.4 µm.

#### 3.1.4. DTA Results

**Figure 4:**
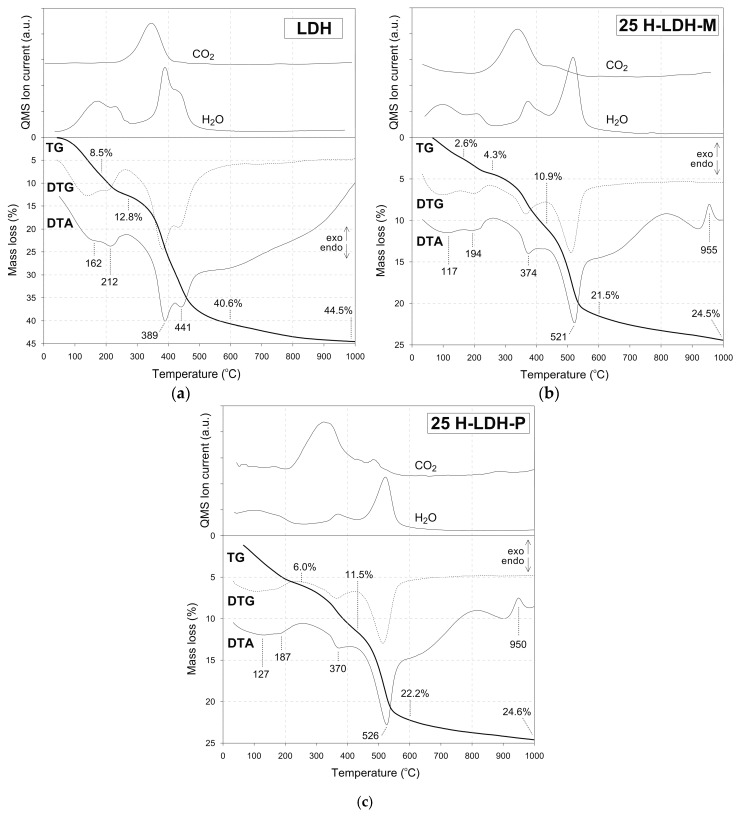
Thermal curves of (**a**) LDH, (**b**) 25 H-LDH-P and (**c**) 25 H-LDH-M.

Thermal curves of the LDH confirmed that Mg/Al LDH was formed during the synthesis (Figure 4) as attested by four characteristic endothermic thermal effects. The first two are attributed to the release of water that took place up to 250 °C with maxima at 162 °C and 212 °C. The corresponding mass losses were equal to 8.5% and 4.3%, respectively. This process was also confirmed by two appropriate H_2_O mass signals. A clear dual effect reveals the presence of water in two different local environments possibly due to the interaction with interlayer anions. The water effects are followed by two signals assigned to decarboxylation and dehydroxylation of the LDH structure in the range of ~380–450 °C with maxima at 389 °C and 441 °C, respectively. The decarboxylation is in particular manifested by a release of CO_2_, followed by H_2_O release recorded in the mass spectrum.

The interpretation of thermal curves for the H-LDH materials showed several similarities in terms of thermal behavior, thus it will be mainly discussed for the 25 H-LDH-M and 25 H-LDH-P samples (Figure 4). In general, the additional presence of halloysite was manifested by appearance of its endothermic dehydroxylation effect with maximum at 525 °C ± 2 °C. This effect was accompanied by an intense H_2_O mass signal. Moreover, at 954 °C ± 3 °C an exothermic peak is visible, which is attributed to the synthesis of new oxide phases from the decomposed aluminosilicate structure. The LDH presence is visible due to its dehydroxylation band with the maximum found in the range of 336–374 °C. The intensity of this effect increases with the increase of LDH content; however, its position is also significantly affected. The dehydroxylation temperature increases with the increase in LDH content from ~337 °C (5 H-LDH-M and 5 H-LDH-P samples) up to ~363 °C (15 H-LDH-M and 15 H-LDH-P samples) and reaches ~372 °C for the 25 H-LDH-M and 25 H-LDH-P samples (Figure 4 and Figure S1). This difference may be attributed to the dispersion of LDH particles in the halloysite matrix. The lower amount of LDH reduces the surface coverage, which subsequently lowers the decomposition temperature. The thermal curves do not show significant differences between the materials prepared by using two different approaches: P and M. The only difference can be seen in the region attributed to water release <200 °C. The curves of materials prepared by the mixing method have an additional signal due to H_2_O release, in particular visible in the mass spectrum which could be due to the different hydration behavior of LDH surface.

By assuming decomposition of dehydrated aluminosilicate Al_2_Si_2_O_5_(OH)_4_ into Al_2_O_3_, 2SiO_2_ and 2H_2_O, the content of halloysite and subsequently LDH was determined. The LDH content was close to the assumed and equal to 6.8% for 5 H-LDH-M and 5 H-LDH-P, 15.4% for 15 H-LDH-M and 15 H-LDH-P, 24.0% for 25 H-LDH-M and 23.3% for 25 H-LDH-P.

### 3.2. Adsorption Experiments

#### 3.2.1. As(V) Removal

**Figure 5:**
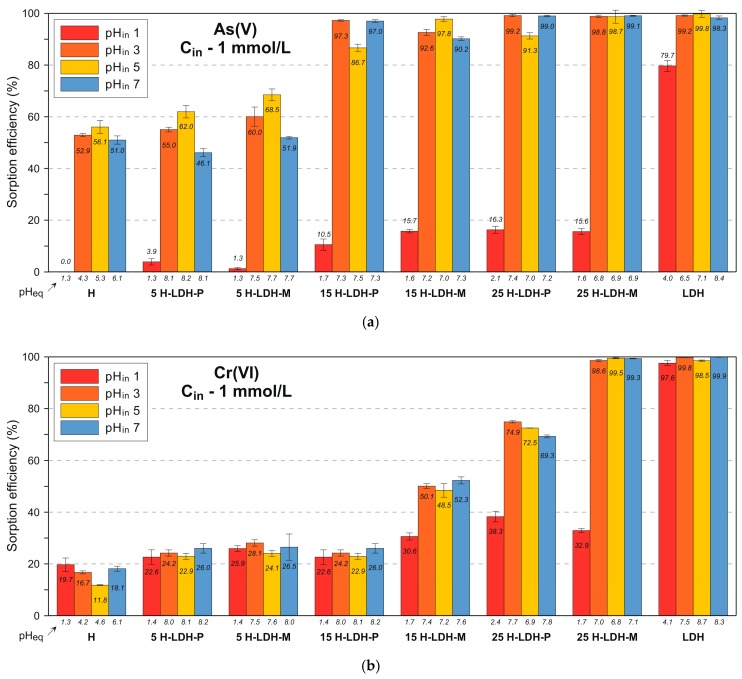
Adsorption efficiency after reaction of the adsorbents with (**a**) As(V) and (**b**) Cr(VI). Initial concentration (C_in_) 1 mmol/L. Error bars may not be visible due to low discrepancy of results.

The removal efficiency of As(V) by the LDH was very high regardless of the C_in_ (Figure 5a and Figure S2a). For the pH_in_ 3, 5, and 7 it exceeded 98%. The As(V) uptake was only lower for the pH_in_ 1 where it reached ~80% (C_in_ 1 mmol/L) and ~68% (C_in_ 5 mmol/L). The halloysite sample also showed a high uptake for the C_in_ 1 mmol/L found in the range of 51%–56% at pH_in_ 3, 5, and 7. In this case the adsorption was below detection at acidic pH_in_ 1. This could be due to the presence of H_3_AsO_4_ that according to the modelled ionic species diagrams dominates over H_2_AsO_4_^−^ and HAsO_4_^2−^ forms at a pH below 2 [46]. The higher C_in_ of 5 mmol/L lowered the adsorption to 9.5%–18.7% (pH_in_ 3, 5 and 7) and it was equal to 3.6% for pH_in_ 1. The pH_eq_ for which the adsorption was the highest in the case of LDH was in the 6.5–8.5 range. These values were lower for the halloysite: 4.0–6.1. At a higher C_in_ of 5 mmol/L a lowering of As(V) uptake was observed with a pH increase for the halloysite. This was related to the lower amount of protonated OH groups at the particle’s edges, which are the main active centers of the kaolin group minerals as well as competition with aqueous OH groups. The removal efficiency in the case of H-LDH materials was very high for the pH_in_ 3, 5 and 7. The samples containing 15 and 25 wt % of LDH removed more than 86% of As(V). Especially the 25 H-LDH samples showed an efficiency very similar to the pure LDH. The adsorption for the 5 H-LDH materials was lower and comparable to the pure halloysite: 46–68%. The results showed that the appropriate content of LDH was crucial for the overall efficiency of the H-LDH materials. The sorption efficiency of the raw LDH and the most efficient 25 H-LDH-M sample calculated for the C_in_ 5 mmol/L corresponded to adsorption capacity equal to 19.5 ± 0.1 mg As/g and 15.3 ± 0.7 mg As/g, respectively. The values were lower than reported in the literature, e.g., 32.6 mg As/g [47] and 54.9 mg As/g [48], however the discrepancy may be due to different experimental conditions and preparation of LDH.

The pH_eq_ was very similar despite different pH_in_ (3, 5, and 7) and was found in the range of 6.8–8.2. The As(V) uptake by the H-LDH materials at acidic pH_in_ 1 was not significant and clearly lower. It did not exceed 16.3%, which was noticed for the 25 H-LDH-P material. This was also due to a low pH_eq_, which was in the range of 1.3–2.1. For the higher C_in_ 5 mmol/L the adsorption capacity of the H-LDH materials was visibly lower; however, a relatively high removal was still observed especially for the 25 H-LDH-M materials: 73–80%. A comparison of the adsorption efficiency for the materials prepared by the two different approaches (P and M) revealed that the M versions showed a slightly higher adsorption especially visible for the higher C_in_.

**Figure 6:**
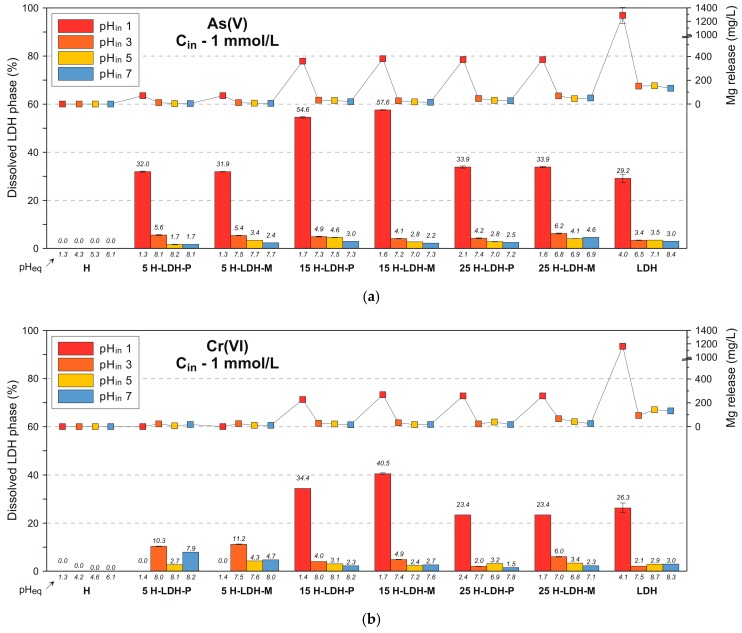
Mg release and percent of dissolved LDH after reaction of the adsorbents: (**a**) As(V) and (**b**) Cr(VI). Initial concentration (C_in_) 1 mmol/L. Error bars may not be visible due to low discrepancy of results.

Although the LDH materials are known as efficient anion exchangers their disadvantage is connected with structural instability at low pH values, which subsequently leads to release of metals which build the brucite-like layers. Thus, the measurement of the Mg(II) concentration was carried out in the supernatant solutions to evaluate the extent of this process (Figure 6a and Figure S3a). As assumed the release of Mg(II) was significant at low pH. The reaction of LDH with aqueous As(V) at pH_in_ 1 was accompanied with Mg(II) release equal to ~1300 mg/L, which corresponds to a dissolution of ~29.2% of the LDH mass. The same phenomena were observed for the LDH that was present in the materials where the Mg(II) concentration was in the 350–500 mg/L range. However, it is worth pointing out that at higher pH_eq_ values (6.8–8.2) the Mg(II) release was much lower for the pure LDH (~30 mg/L) and the materials 5 H-LDH (~10 mg/L), 15 H-LDH (15–20 mg/L) and 25 H-LDH (40–50 mg/L). These values corresponded to a percentage dissolution of LDH mass not higher than ~6%–8%. For the higher As(V) concentration (C_in_ 5 mmol/L) the dissolution was observed at the same level than for the C_in_ 1 mmol/L. This was with exception of the 5 H-LDH materials where dissolution was more pronounced probably due to the lower amount of LDH and thus greater susceptibility of individual particles for protons.

#### 3.2.2. Cr(VI) Removal

The removal efficiency of Cr(VI) by the pure LDH was very high (>97.6%) regardless of the C_in_ and pH_in_ (Figure 5b and Figure S2b). The pH_eq_ values found in the range of 4.0–8.7 confirm a negligible impact of pH on adsorption in this case. The Cr(VI) adsorption by the pure halloysite sample was lower than in the case of As(V) and did not exceed 19.7%. However, in contrast to the reaction with As(V) here a visible uptake of Cr(VI) was also evidenced for the pH_in_ 1. This can be explained by differences in formed ionic species. For As(V) the H_3_AsO_4_ starts to dominate at a pH below 2 while Cr(VI) mainly exists as HCrO_4_^−^ and Cr_2_O_7_^−^ with very low probability for H_2_CrO_4_ formation [46]. The removal of Cr(VI) by the materials was in general less efficient than that observed for As(V) due to the less efficient contribution of the halloysite component. For the 25 H-LDH materials the removal reached ~40%–43% and a decrease of uptake was noticed with the lower LDH content: 15 H-LDH (not higher than 31.3%) and 5 H-LDH (not higher 21.2%). The M version of the halloysite-LDH material (25 H-LDH-M) showed a visibly higher adsorption for the lower C_in_. The adsorption capacity of the raw LDH and the 25 H-LDH-M, calculated for the C_in_ 5 mmol/L, was equal to 12.7 ± 0.2 mg Cr/g and 5.9 ± 0.5 mg Cr/g, respectively. The value for the raw LDH was found in the range of previously reported capacity of 17 mg Cr/g [49], 16.3 mg Cr/g [50] and 9.0 mg Cr/g [51]. The dissolution of LDH during adsorption of Cr(VI) showed similar trends as in the case of As(V), however the overall Mg(II) release and percentage of dissolved LDH were lower (Figure 6b and Figure S3b).

### 3.3. Solid State Analysis after Adsorption and Insight into Removal Mechanisms

**Figure 7:**
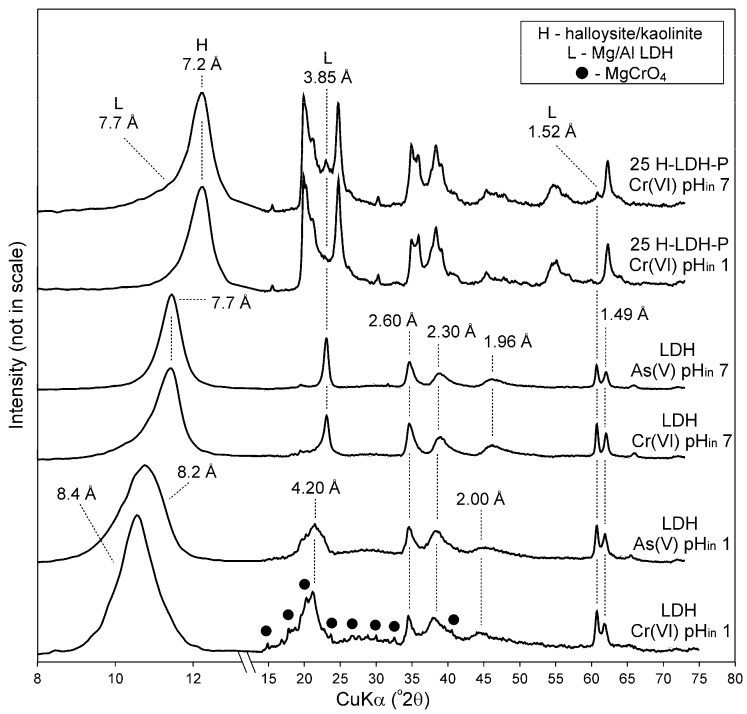
XRD pattern of selected samples after reaction with As(V) and Cr(VI).

**Figure 8:**
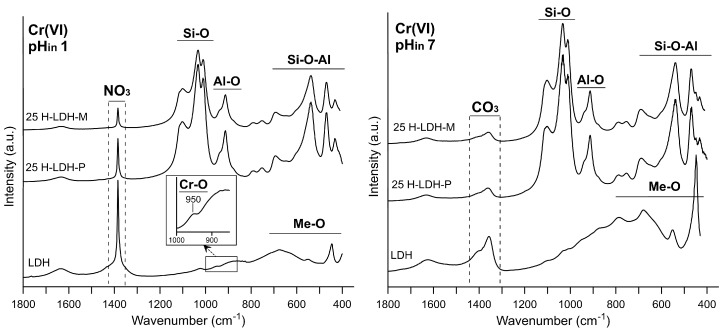
FTIR spectra of selected samples after reaction with Cr(VI).

The XRD patterns of the LDH sample showed significant changes after adsorption of As(V) and Cr(VI) at pH_in_ 1 (Figure 7). The 003 and 006 reflections were broadened and shifted to higher values, which indicated a loss of crystallinity through partial dissolution at low pH. This was also confirmed by FTIR spectra where the bands attributed to the metal–oxygen framework are less resolved and intense in comparison to the spectra of LDH after reaction at pH_in_ 7 (Figure 8 and Figure S4). Moreover, the broadening of the XRD reflections was due to structure delamination induced by nitrates intercalation [36,52]. Regardless of the material at pH_in_ 1, a sharp band attributed to nitrates was observed after As(V) and Cr(VI) at 1385 cm^−1^. The presence of nitrates was due to HNO_3_, which was used for pH adjustments. For the LDH sample after Cr(VI) adsorption at pH_in_ 1 characteristic reflections of magnesium chromate (ICDD #01-243) MgCrO_4_ were identified. This was also attested by the presence of an additional band at 950 cm^−1^ ascribed to magnesium chromate [53]. The precipitation mechanism was an effect of the Cr(VI) reaction with Mg released from the LDH. The XRD patterns of 25-H-LDH samples showed that LDH reflections after As(V) and Cr(VI) adsorption disappeared, or were less resolved, which indicated LDH partial dissolution at pH_in_ 1. The changes of the XRD patterns were not observed for the samples after adsorption at pH_in_ 7, which confirmed that ion-exchange in the case of the LDH component dominated as the removal mechanism [54,55]. Moreover, chemisorption by halloysite contributed to As(V) removal as shown in earlier studies [2,56]. The contribution of the LDH edge sites and their susceptibility to form inner-sphere complexes with oxyanions also cannot be fully excluded [57]. Despite high adsorption of As(V) or Cr(VI) the FTIR spectra indicated the dominating presence of interlayer carbonates as evidenced by two bands with maxima at 1360 and 1385 cm^−1^.

**Figure 9:**
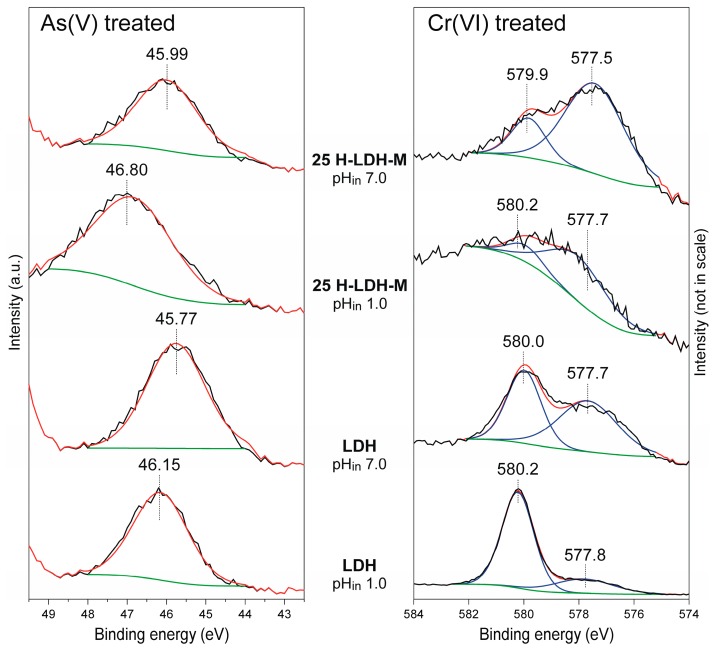
XPS spectra (As 3d and Cr 2p_3/2_) of LDH and 25 H-LDH-P after reaction with As(V) and Cr(VI). Initial concentration (C_in_) 5 mmol/L.

The XPS method was used to get insight into possible oxidation state changes of As and Cr after adsorption (Figure 9). Regardless of the sample in the case of As, the As 3d spectra revealed one peak with a maximum found in the range of 45.8–46.8 eV. This peak can be solely attributed to the As(V) oxidation state and confirms that reduction did not take place in the applied conditions [58]. The difference in the peak position can be related to protonation of As ionic species [58]. At a low pH_in_ of 1 the values were shifted towards higher eV as a result of protonation and subsequent formation of H_2_AsO_4_^−^, which was more pronounced for the 25 H-LDH-M material. In turn, at a high pH_in_ of 7 the lower binding energy values indicated the presence of less protonated HAsO_4_^2−^ species. For the Cr-treated samples clear evidences for the Cr(VI) reduction were found in the Cr 2p spectra. The fitted Cr 2p_3/2_ peak showed components attributed both to Cr(VI) and Cr(III) in the range of 579.9–580.2 eV (FWHM 1.4–1.5 eV) and 577.5–577.8 eV (FWHM 2.6 eV), respectively [59,60]. Clearly the reduction of Cr(VI) was influenced by pH_eq_. At a low pH_in_ of 1 the Cr(III) component had a lower intensity, indicating less efficient reduction. On the other hand, the intensity of this component increased with pH. The Cr(VI) to Cr(III) components ratio suggested that reduction was more efficient for the 25 HLDH-M sample as compared to LDH, which could be due to the Fe presence in the H sample.

## 4. Conclusions

The Mg/Al LDH was synthesized successfully on the halloysite surface, using both direct precipitation and physical mixing approaches, which was confirmed by XRD. The SEM images revealed LDH layered particles surrounding the characteristic tubular forms of the halloysite. The FTIR and DTA analyses showed the dominating presence of carbonates in the interlayer space of the LDH. The raw LDH phases exhibited high adsorption efficiency towards both As(V) and Cr(VI) for a pH_in_ in the range of 3–7. The halloysite-LDH materials revealed satisfying adsorption efficiency in comparison to the LDH. The XRD and chemical analysis after adsorption experiments confirmed partial dissolution of the LDH, which resulted in a low adsorption efficiency at pH_in_ 1. As a consequence of LDH dissolution, the evidence for MgCrO_4_ precipitation was found in the XRD and FTIR analyses. The XPS analysis of Cr 2p spectra of both LDH and halloysite-LDH materials confirmed a partial reduction of Cr(VI) to Cr(III), mostly at pH_in_ 7. Beside precipitation and reduction in the case of Cr(VI), the anion exchange mechanism took place for both As(V) and Cr(VI). The application of halloysite-LDH materials allowed for efficient As(V) and Cr(VI) removal even at very high C_in_ (5 mmol/L). It is worth underlining that the final cost of adsorbents can be significantly reduced by an application of halloysite-LDH materials instead of the raw LDH phase, making it economically viable. Moreover, the preparation of such materials in granulated form can reduce the swelling properties of LDH and allow its future application in column flow systems.

## Supplementary Materials

The following are available online at https://www.mdpi.com/1996-1944/12/21/3569/s1, Figure S1: Thermal curves of the materials: (**a**) 5-H-LDH-M, (**b**) 5 H-LDH-P, (**c**) 15 H-LDH-M, (**d**) 15 H-LDH-P. Figure S2: Adsorption efficiency after reaction of the adsorbents with (**a**) As(V) and (**b**) Cr(VI). Initial concentration (Cin) 5 mmol/L. Error bars may not be visible due to low discrepancy of results. Figure S3: Mg release and percent of dissolved LDH after reaction of the adsorbents: (**a**) As(V) and (**b**) Cr(VI). Initial concentration (Cin) 5 mmol/L. Error bars may not be visible due to low discrepancy of results. Figure S4. FTIR spectra of selected samples after reaction with As(V).
