# Supplementary material for: Performance of Halloysite-Mg/Al LDH Materials for Aqueous As(V) and Cr(VI) Removal
Source: Materials (Basel). 2019 Oct 31;12(21):3569. doi: 10.3390/ma12213569 (PMC6862184; doi:10.3390/ma12213569)
Supplement: Supplementary file 1 [file materials-12-03569-s001.pdf]

materials

*Supplementary Information*

# Performance of Halloysite-Mg/Al LDH Materials for Aqueous As(V) and Cr(VI) Removal

**Jakub Matusik [1,*], Jakub Hyla [1], Paulina Maziarz [1], Karolina Rybka [1] and Tiina Leiviskä [2]**

[1] Department of Mineralogy, Petrography and Geochemistry , Geophysics and Environmental Protection, Faculty of Geology, AGH University of Science and Technology, Mickiewicza 30, 30-059 Krakow, Poland; kuba.251@wp.pl (J.H.); pmaziarz@agh.edu.pl (P.M.); krybka@agh.edu.pl(K.R.)

[2] Chemical Process Engineering, University of Oulu, P.O. Box 4300, FIN-90014 University of Oulu, Oulu, Finland; tiina.leiviska@oulu.fi

* Correspondence: jmatusik@agh.edu.pl; Tel.: +48-126175233

Received: 24 September 2019; Accepted: 30 October 2019; Published: date

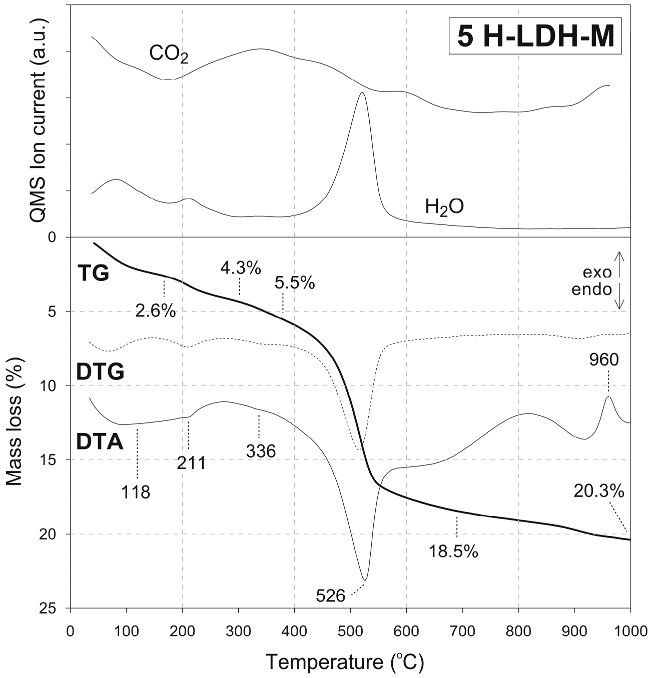


(a)

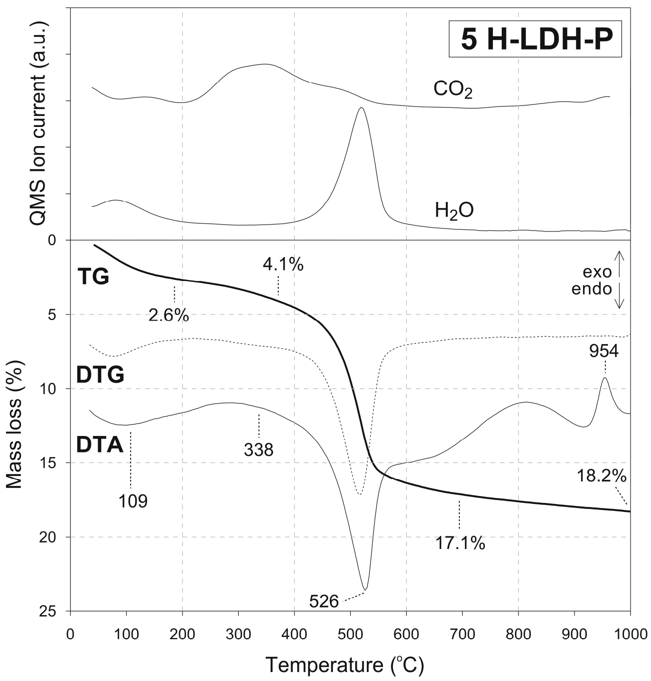
5 H-LDH-P
QMS Ion current (a.u.)
$CO_2$
$H_2O$
TG
DTG
DTA
exo
endo
4.1%
2.6%
954
338
109
18.2%
17.1%
526
Mass loss (%)
Temperature (°C)

(**b**)

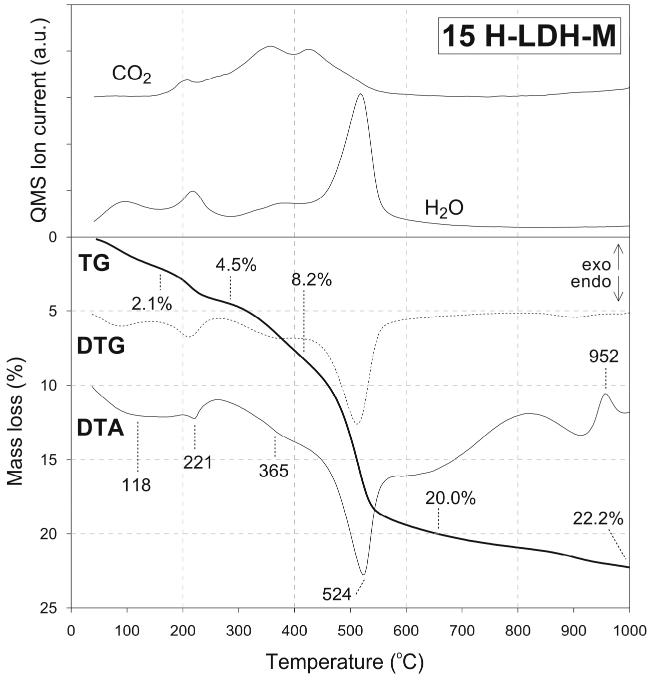
15 H-LDH-M
QMS Ion current (a.u.)
$CO_2$
$H_2O$
TG
DTG
DTA
exo
endo
4.5%
8.2%
2.1%
952
118
221
365
20.0%
22.2%
524
Mass loss (%)
Temperature (°C)

(**c**)

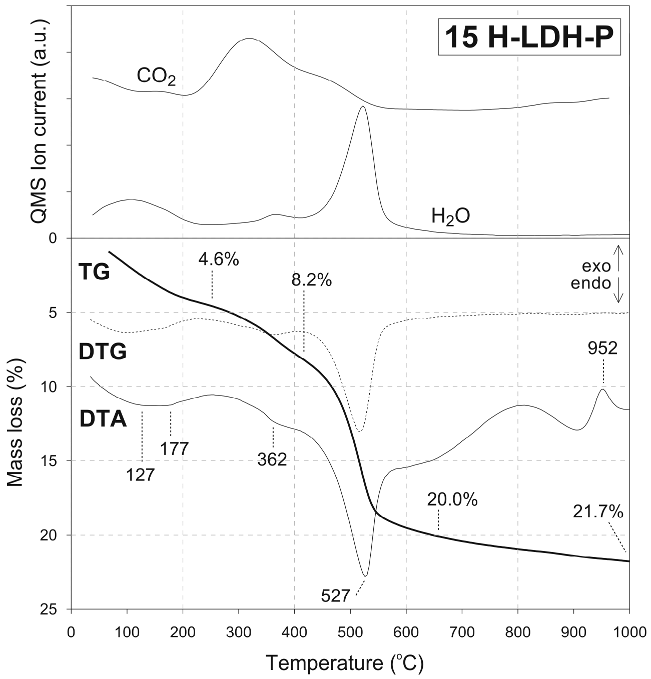


(**d**)

**Figure S1.** Thermal curves of the materials: (**a**) 5-H-LDH-M, (**b**) 5 H-LDH-P, (**c**) 15 H-LDH-M, (**d**) 15 H-LDH-P.

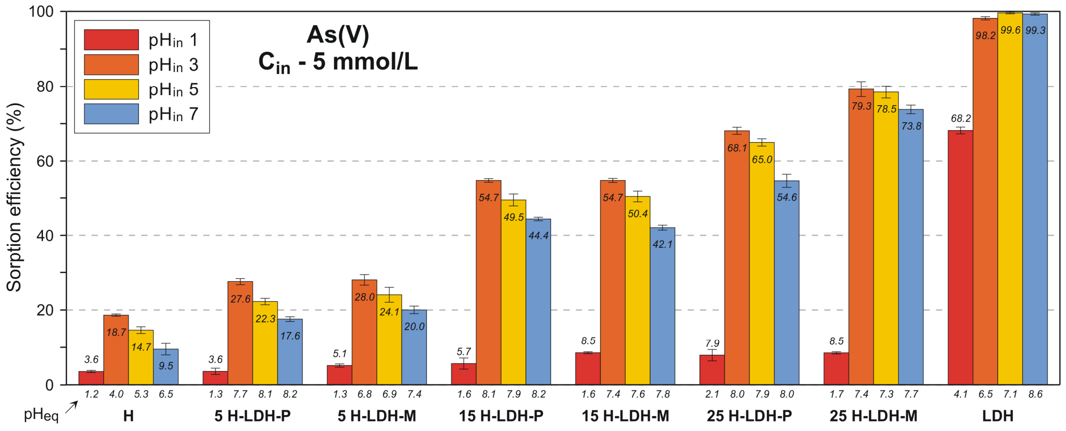


(**a**)

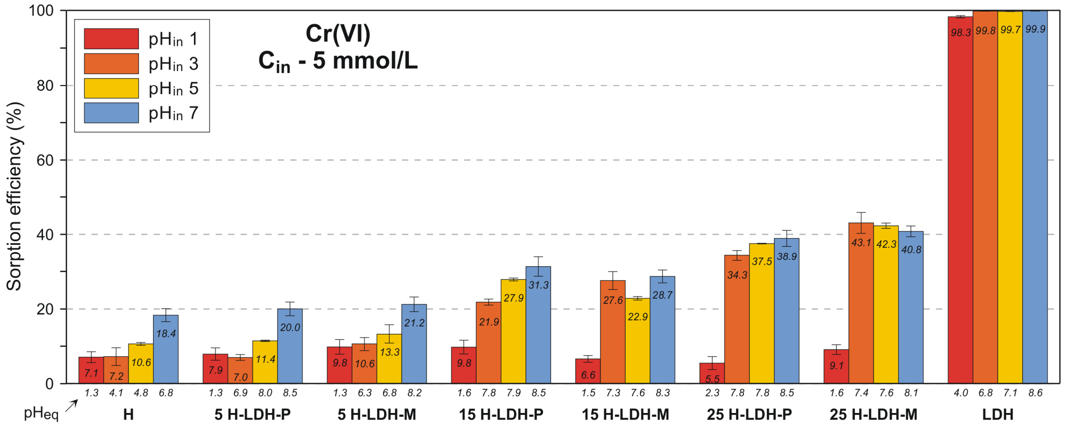


(**b**)

**Figure S2.** Adsorption efficiency after reaction of the adsorbents with (**a**) As(V) and (**b**) Cr(VI). Initial concentration (Cin) 5 mmol/L. Error bars may not be visible due to low discrepancy of results.

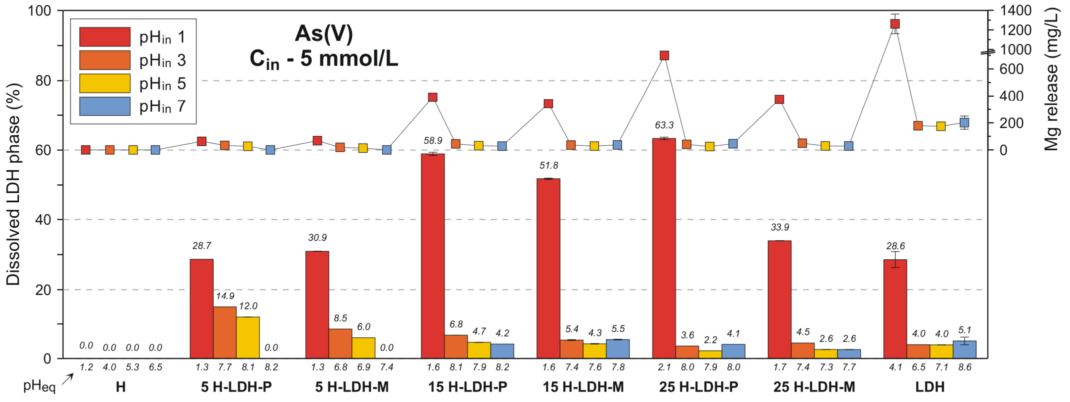


(**a**)

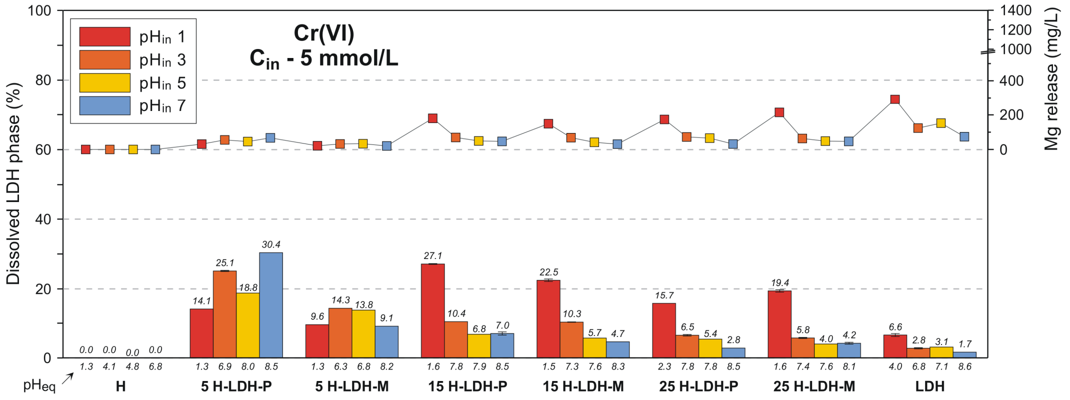


(**b**)

**Figure S3.** Mg release and percent of dissolved LDH after reaction of the adsorbents: (**a**) As(V) and (**b**) Cr(VI). Initial concentration (Cin) 5 mmol/L. Error bars may not be visible due to low discrepancy of results.

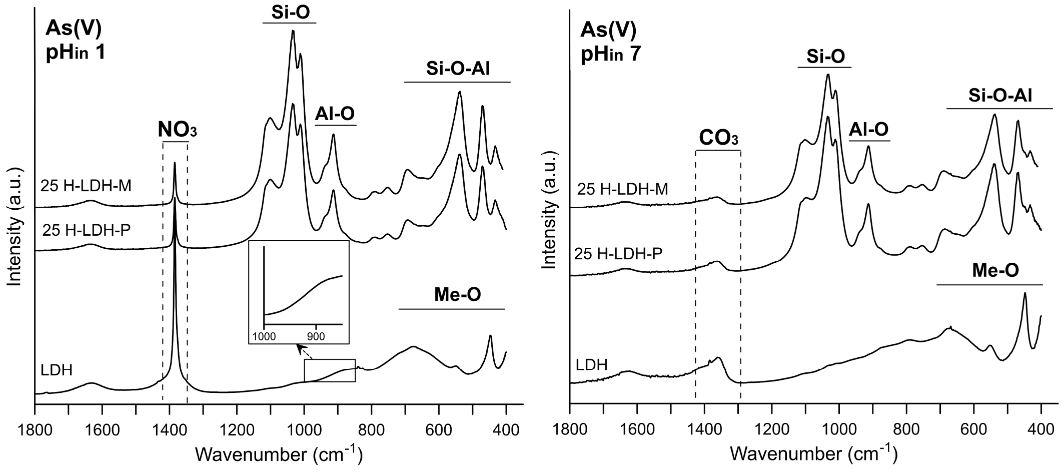


**Figure S4.** FTIR spectra of selected samples after reaction with As(V).

© 2019 by the authors. Submitted for possible open access publication under the terms and conditions of the Creative Commons Attribution (CC BY) license (http://creativecommons.org/licenses/by/4.0/).
